# Toxicity and Anti-promastigote Activity of Benzoxazinoid Analogs Against* Leishmania (Viannia) braziliensis* and *Leishmania (Leishmania) infantum*

**DOI:** 10.15171/apb.2020.015

**Published:** 2019-12-11

**Authors:** Gilberto de Sousa, William Gustavo Lima, Flávio José dos Santos, Francisco A. Macías, José María González Molinillo, Rafael Gonçalves Teixeira-Neto, João Máximo de Siqueira, Eduardo Sérgio da Silva

**Affiliations:** ^1^Laboratório de Parasitologia e Doenças Parasitárias, Campus Centro-Oeste Dona Lindu, Universidade Federal de São João Del-Rei (UFSJ), Divinopolis, MG, Brazil.; ^2^Laboratório de Microbiologia Médica, Campus Centro-Oeste Dona Lindu, Universidade Federal de São João Del-Rei (UFSJ), Divinopolis, MG, Brazil.; ^3^Laboratório de Farmacognosia/Química de Produtos Naturais, Campus Centro-Oeste Dona Lindu, Universidade Federal de São João Del-Rei (UFSJ), Divinopolis, MG, Brazil.; ^4^Allelopathy Group, Department of Organic Chemistry, Institute of Biomolecules (INBIO), Campus CEIA3, School of Science, University of Cadiz, Puerto Real (Cádiz), Spain.

**Keywords:** Benzoxazinone core, Leishmanicidal agents, Neglected disease, Pharmacology, Pyridoxazinone core, Splenic hamster cells

## Abstract

***Purpose:*** Here, we aim to evaluate the antileishmanial activity of compounds with a benzoxazinoid (BX) skeleton, previously synthesized by our group, against *Leishmania (Viannia) braziliensis* and *Leishmania (Leishmania) infantum* promastigotes.

***Methods:*** Anti-promastigote activity, as well as cytotoxicity, were determined using the 3-(4,5-dimethylthiazol-2-yl)-2,5-diphenyltetrazolium bromide (MTT) colorimetric assays. The selectivity index (SI) for each compound was calculated using a ratio of the cytotoxicity of compounds and the geometric mean (GM) of antileishmanial concentrations to each species tested. The comparisons between groups were carried out using a t test or analysis of variance (one-way ANOVA). A P value of less than 0.05 was considered significant.

***Results:*** All the compounds tested were active, with IC_50_ falling between 92±6.19 µg/mL and 238±6.57 µg/mL for L. braziliensis, and 89±6.43 µg/mL and 188±3.58 µg/mL against *L. infantum*. Bex2, Bex3, Pyr1, Pyr2, and Pyr4 were compounds that showed activity similar to the drug Glucantime®, exhibited low cytotoxicity against splenic hamster cells (CC50 raging between >400 and 105.7±2.26 µg/mL) and had favorable selectivity indices (SI 1.12 to 3.96).

***Conclusion:*** The analogs in question are promising prototypes for the pharmaceutical development of novel, safer and more effective leishmanicidal agents.

## Introduction

Leishmaniases are zoonoses that manifest in the cutaneous, mucocutaneous and visceral forms.^1^ Among all parasitic infections, leishmaniasis, which is related to 20 000–30 000 deaths annually, ranking second in mortality after malaria.^1,2^ Leishmaniasis is a vector-borne, infectious disease endemic to 98 countries. Transmission occurs through the bite of a naturally infected *Phlebotomus* in the Old World or *Lutzomyia* in the New World.^3^ Cutaneous leishmaniasis (CL) is the most common type, with an estimated 0.6–1.0 million new cases occurring each year, corresponding to 75% of all diagnosed cases of leishmaniasis.^1^ The cutaneous disease is mainly caused by subgenus *Leishmania* (e.g., *L. major, L.tropica, L.mexicana, L.amazonensis*) in the Middle East, Asia, and Africa, while the subgenus *Viannia* (*L.naiffi, L.braziliensis, L.guyanensis, L.panamensis)* is responsible for most cases occurring in the Americas.^4^ More than 90% of CL cases occur in Afghanistan, Algeria, Brazil, Colombia, Iran and Syria.^1^ In Brazil, the Ministry of Health, through the National Health Information System, has estimated that the incidence rate of CL in the country is 11.1 cases/100 000 inhabitants.^5^

Visceral leishmaniasis (VL; also known as the kala–azar disease) is caused by *L. (L.) infantum*, *L.chagasi*, or *L.donovani* complexes.^6^ Although less frequent, VL affects 50 000 to 90 000 individuals annually and is the most severe form of the disease. It is characterized by fever, pancytopenia, thrombocytopenia, hepatosplenomegaly, pain, and abdominal distention, wasting and malnutrition.^1,6^ Currently, particular attention has been given to this type of leishmaniasis due to significant changes the transmission cycle of the disease, which has become increasingly urbanized and anthropophilic.^7^ In 2015, Brazil, Ethiopia, India, Kenya, Somalia, South Sudan, and Sudan accounted for more than 90% of cases of VL,^1^ and between 2000–2010, an average of 3484 new cases each year were reported in Brazil.^7^

The therapeutic arsenal used to leishmaniases is basically composed by pentavalent antimony, paromomycin, pentamidine, miltefosine, and amphotericin B.^8^ However, these drugs have several limitations which include severe side effects (e.g., cardiac arrhythmias, myalgia, and pancreatitis associated to pentavalent antimonial), high toxicity, and require prolonged use. In addition patients in need of treatment exhibit a low adherence to treatment plans and there is an insufficient availability of drugs generally in endemic regions.^9^ Additionally, *Leishmania* strains with moderate and complete resistance to pentavalent antimonials and amphotericin B have already been identified in several parts of the world.^10^ Problems with available therapies are even more pressing the pharmaceutical industry has neglected to investment in the research and development of new compounds with antileishmanial activity; a class of drugs that has represented less of 1% of drugs researched in the last year by the most prominent companies.^11^ Thus, the search for new pharmaceutical compounds is urgently needed to control leishmaniasis.

Currently, the World Health Organization (WHO) recommended the use of some herbal medicines as antileishmanial agents and several studies have determined that the phytoconstituents of these medicinal plants should be considered prototypes for new leishmanicidal drugs.^1,9^ Thus, the benzoxazinoids (Bexs), which are allelochemical alkaloids widely distributed among monocotyledons of the Gramineae family, stand out as a potential targets in the search for new antileishmanial agents.^12^ Bexs are characterized by the presence of a 2-hydroxy-*2H*-1,4-benzoxazin-3(*4H*)–one skeleton and are known to show anti-inflammatory, antinociceptive, reproductive and immune stimulatory, antitumor and appetite suppressor activities.^12,13^ Moreover, recent work has uncovered a critical antimicrobial activity for the compounds, covering bacteria, yeast, molds, and retroviruses of great medical importance.^14-17^

Our group has previously developed and synthesized a series of new Bex analogs that have shown antimicrobial activity against microorganisms of high medical relevance such as *Enterococcus faecalis*, *Staphylococcus aureus*, *Acinetobacter baumannii*, *Candida albicans*, *C. tropicalis* and *C. glabrata*.^18^ Here, we aimed to evaluate potential antileishmanial effects of these compounds against promastigotes of *Leishmania (V.) braziliensis* and *Leishmania (L.) infantum* and also aim determine the cytotoxicity of these analogs against hamster spleen cells.

## Materials and Methods

### Chemistry

**Figure 1:**
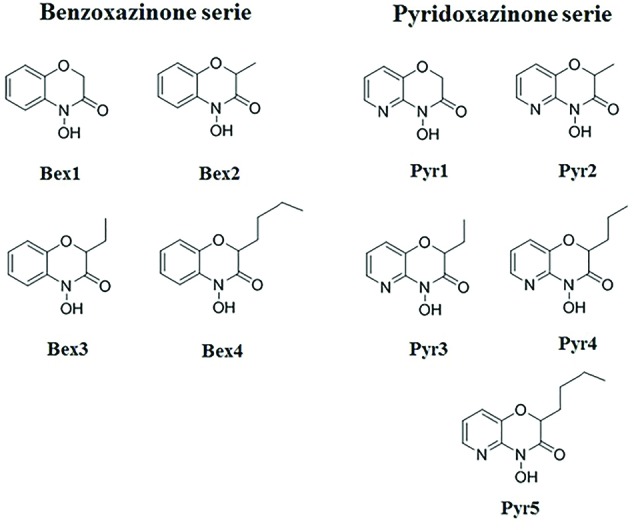
Chemical structures of benzoxazinoids analogs employed in this study. Pyr1-5: Pyridoxazinone series; Bex1-5: Benzoxazinone series.

The Bexs analogs employed in this study were provided by the Laboratory of Natural product chemistry, Federal University of Sao Joao Del-rei, Divinópolis, MG, Brazil. These compounds were synthesized according to the methodology described by Lima et al,^18^ and chemical structures of the compounds are shown in Figure 1.

### Parasite culture

Promastigotes of *Leishmania (V.) braziliensis* (MHOM/BR/75/M2903) and *Leishmania (L.) infantum* (MHOM/BR/74/PP75) were grown in a biphasic medium. The solid phase was composed of Nicolle–Novy–MacNeals (NNN) medium with 15% defibrillated rabbit blood. Already in the liquid phase, we employed Roswell Park Memorial Institute (RPMI)-1640 (Sigma-Aldrich, St. Louis, USA) or Liver Infusion Tryptose (LIT) medium with 15% fetal bovine serum (Gibco – Thermo Fisher Scientific, Waltham USA), both supplemented with 1% of penicillin-streptomycin antibiotic solutions (10.000 U/mL of penicillin and 10.000 μg/mL of streptomycin; Gibco – Thermo Fisher Scientific, Waltham, USA). Cultures were maintained in an incubator at 25°C until parasites reached logarithmic phase (4 days). Next, the cultures were centrifuged and the pellet was suspended in RPMI-1640 or LIT for counting promastigotes using a Neubauer hemocytometer under an optical microscope (Axiostar Plus Carl Zeiss, Jena, Germany) at 400x magnification. Subsequently, parasites were diluted in RPMI-1640 or LIT medium at a final concentration of 1 x 10^6^parasites/mL for use in antileishmanial assays.

### Anti-promastigote activity

The 50% inhibitory concentration (IC_50_) against *L. (V.) braziliensis* and *L. (L.) infantum* was evaluated by determination of the parasite viability using the colorimetric 3-(4,5-dimethylthiazol-2-yl)-2,5-diphenyltetrazolium bromide (MTT) method.^19^ Initially, 100 μL of the promastigote suspension prepared in RPMI-1640 or LIT medium (1 × 10^6^cells/mL) was added in each well of the 96-well microplate. Parasites were then treated with two-fold serial dilutions (from 3.12 to 400 µg/mL) of Bex-derived analogs prepared in 10% dimethyl sulfoxide (DMSO) (Sigma-Aldrich, St. Louis, USA) and the microplates were incubated at 25°C for 48 h. Next, 20 μL of MTT solution at 5 mg/mL (Sigma-Aldrich, St. Louis, USA) was added to the parasite cultures and the plates were covered with laminated paper and incubated at 37°C for 4 h. Finally, the plates were centrifuged at 1500 rpm for 15 min at 4°C (Heraeus-Megafuge 11R Centrifuge - Thermo Fisher Scientific, Waltham USA) and the formazan crystals formed were dissolved by the addition of 200 μL 10% DMSO. After further incubation for 5 min at room temperature, absorbance at 570 nm of samples measured using a microplate reader (Metller Toledo, Brazil). Meglumine antimoniate (Glucantime^®^Sanofi-aventis, France) and amphotericin B (Eurofarma, Brazil) were included as positive controls, and 10% DMSO solution was used as a solvent control. Samples containing RPM1-1640 or LIT only were used to control the sterility of media and samples containing inoculum and medium, but not compounds, were employed as growth controls.

### Cell culture and cytotoxicity assays

Hamster spleen cells were used to test cytotoxicity. The cells were kindly provided by Dr. Rosy Iara Maciel de Azambuja Ribeiro from of Laboratory of Experimental Pathology/UFSJ. The cells were cultivated in saline solution (NaCl 0.9%; Synth, Brazil) and maintained at 37°C in an incubator with an atmosphere containing 5% CO_2_ (Thermo Fisher Scientific, Waltham, USA).

Cytotoxic concentration determinations for 50% of the cells in culture (CC_50_) were evaluated by using the colorimetric 3-(4,5-dimethylthiazol-2-yl)-2,5-diphenyltetrazolium bromide (MTT) method, following protocols outlined in a previous study.^19^ Briefly, spleen cells were exposed for 48h to several concentrations of compounds (3, 6.25, 12.5, 25, 50, 100, 200, 400 and 800 µg/mL) and, afterward, the MTT assay was used to quantify the cell viability as was done using the anti-promastigote assay. Finally, CC_50_ values were calculated by linear regression analysis of absorbance values obtained previously.^20^

### Selective index (SI) determination

The selectivity of Bex analogs and antileishmanial agents was determined to evaluate the safety of compounds. Thus, the selectivity index (SI) was calculated through the ratio between the CC_50_ of compounds against the hamster spleen cells and the geometric mean (GM) of IC_50_ values calculated for *L. (V.) braziliensis* and * L. (L.) infantum*.^21^ In the case where CC_50_ was higher than the highest concentration tested of the compound, to calculate the SI, the value used was 400. The larger SI was indicative of a safer molecule.

### Statistical analysis

All experiments were performed in triplicate and with at least three independent experimental repetitions. Results are presented as mean ± standard error of these repetitions as appropriate. In our statistical analysis, the normality of the data was initially evaluated using the Shapiro-Wilk test. Subsequent comparisons between the groups were carried out using a t-test (two groups) or analysis of variance (one-way ANOVA) (multiple groups). The Tukey test was used to compare the results of the treatments and the Dunnett test to compare the results of the treatment and control. A *p*-value of less than 0.05 was considered significant. Statistical analysis was performed using GraphPad Prism (version 6) (Graphpad Software Inc., San Diego, CA, USA).

## Results and Discussion

Bexs are alkaloids, characterized in the 1950s, that are commonly found in monocotyledons of the Gramineae family, such as rye (*Secalecereale*), maize (*Zeamays*) and wheat (*Triticumaestivum*).^12^ Since then, appreciable amounts of Bexs have also been reported in whole food products,^12^ as well as in some dicotyledons of the families Acanthaceae (i.e., *Acanthusmollis, Aphelandratetragona, A.squarrosa,Blepharisedulis*), Ranunculaceae (*Consolidaorientalis*), Plantaginaceae (*Scopariadulcis*), Lamiaceae (*Lamiumgaleobdolon*) and Scrophulariaceae.^22^ The Bex-derived stand out for their importance as allelochemicals, used for plant-plant, plant-insect, or plant-microorganisms communication (or interaction), and display a wide range of anti-infective, insecticidal and antimicrobial activities.^12,13,23^

In addition to the defensive effect against phytopathogens, Bex-derived compounds have also shown activity against microorganisms of medical interest.^14-17^ Bravo et al^15^ reported that the Bexs benzoxazolin-2-one (BOA), 2-hydroxy-1,4-benzoxazin-3-one (HBOA) and 2,4-dihydroxy-1,4-benzoxazin-3-one (DIBOA) exhibit antibacterial effects against *Staphylococcus aureus* and *Streptococcus mutans* at concentrations of 500 μg/mL, and against *Escherichia coli* at a concentration of 1,000 μg/mL according to microdilution broth assays. The compound, 2,4-dihydroxy-7-methoxy-1,4-benzoxazin-3-one (DIMBOA; at a concentration of 2 mg/disk), is active against the bacteria *Enterococcus faecalis*, *Klebsiella pneumoniae*, *Pseudomonas aeruginosa*, *Bacillus subtilis*, *S. aureus* methicillin-resistant and *E. coli* extended-spectrum β-lactamases produced by disc diffusion.^17^ In addition, the antifungal activity of Bex-derived compounds and their metabolites have been recognized against the yeasts *Candida albicans, Candida glabrata*, and *Saccharomyces cerevisae*, and also against molds, *Penicillium chrysogenum*, *Monilia Formosa*, *Phanerochaete chrysosporium* and *Trichoderma reesei*.^12,14,17^ Wang and Ng^16^ showed that Bexs also have antiviral properties; 6-methoxy-benzoxazolin-2-one (MBOA) is able to inhibit reverse transcriptase from human immunodeficiency virus(HIV). Thus, Bexs have proven to be potential sources for the development of novel anti-infective agents. However, the antiparasitic effect of this class is not understood. Various problems associated with the current antileishmanial therapy, i.e., high toxicity, serious adverse effects, long course, storage, and access difficulties; together with the medical, epidemiological and social importance of this zoonosis, highlights the need to study the potential antiparasitic of Bex-derived compounds against the genus *Leishmania*.^1,7-9^ Here, a set of C-2 substituted Bexs, synthesized using the pyridoxazinone and benzoxazinone core as described by Lima et al,^18^ were evaluated for anti-promastigote activity against *L. (V.) braziliensis* and *L. (L.) infantum*.

**Figure 2:**
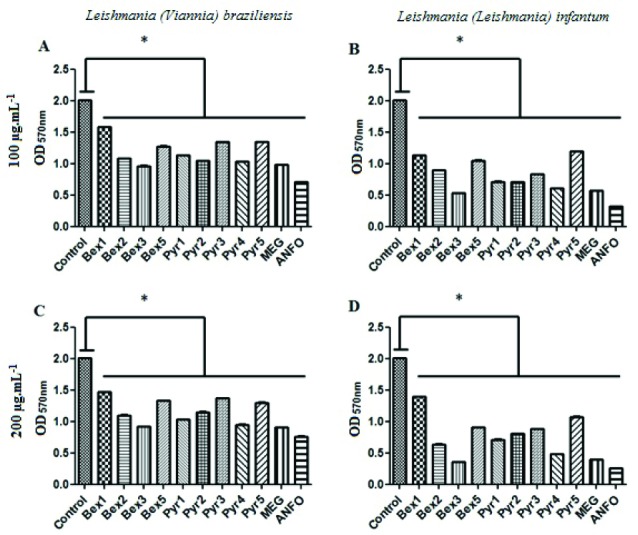
Anti-promastigote activity of benzoxazinoids analogs and positive controls at concentrations of 100 µg/mL (A and B) or 200 µg/mL (C and D) against *Leishmania (Viannia) braziliensis* (A and C) and *Leishmania (Leishmania) infantum* (B and D). ANFO: Amphotericin B; MEG: Meglumine antimoniate (Glucantime^®^); OD_570nm_: Optical density at 570 nm; Pyr1-5: Pyridoxazinone series; Bex1-5: Benzoxazinone series. *Difference between treated samples and untreated controls, One-Way ANOVA with Dunnett pots-test (*P*< 0.05).

As shown in Table 1, all compounds tested were active, with IC_50_ values falling between 92±6.19 µg/mL and 238 ± 6.57 µg/mL against *L. (V.) braziliensis*, and from 89 ± 6.43 µg/mL to 188 ± 3.58 µg/mL against *L. (L.) infantum*. In the concentration of 100 or 200 µg/mL, for example, all compounds, including the positive controls, were able to significantly reduce the viability of *L. (V.) braziliensis* and *L. (L.) infantum* promastigotes compared to untreated controls (Dunnett, *P*< 0.05) (Figure 2). The compounds Bex2, Bex3, Pyr1, Pyr2 and Pyr4 showed a similar to the activities of the drug Glucantime^®^, which is considered the first-line of defense against leishmanial disease.^9^ These compounds, from the concentration of 25 μg/mL, did not differ statistically in the antileishmanial effect when compared to the reference drug. The presence of the pyridine ring, although not a significant influence in Bex activity has been shown to be an essential pharmacophoric moiety in other classes of compounds with leishmanicidal potential, such as chalcones,^24^ pyrazoles,^25^ and dyarylimidazoles.^26^ In addition, the Bexs employed in this study had similar effects on the promastigotes of *L. (V.) braziliensis* and *L. (L.) infantum*, suggesting that they may be used in chemotherapy of both, CL and VL.

**Table 1:** Effect of benzoxazinoids analogs on the growth of *Leishmania (Viana) braziliensis* and *Leishmania (Viana) infantum* promastigotes

**Compounds**	**IC**_50_ **(µg/mL)**			
	***L.*** ***braziliensis*** ^a^		***L.*** ***infantum*** ^a^	
	**LIT** ^b^	**RPMI-1640** ^b^	**LIT** ^b^	**RPMI-1640** ^b^
Bex1(C)	212±6.04	238±6.57	174±3.39	188±3.58
Bex2 (H)	103±4.36	109±4.44	106±5.61	111±5.69
Bex3(G)	92±6.19	97±6.24	89±6.43	93±6.48
Bex5 (E)	129±4.61	138±4.76	141±5.36	151±5.57
Pyr1(A)	108±5.91	115±6.04	99±5.61	103±5.64
Pyr2 (B)	100±5.18	105±5.23	112±5.34	118±5.45
Pyr3 (I)	141±5.94	152±6.17	149±6.35	160±6.62
Pyr4 (D)	98±5.94	103±5.99	91±6.00	95±6.05
Pyr5 (F)	142±3.67	154±3.85	135±4.13	143±4.25
MEG	97±4.34	98±4.45	97±3.14	102±3.54
ANFO	33.3±3.66	33.4±3.01	41.64±4.27	41.45±0.99

IC_50_: 50% inhibitory concentration; MEG: Meglumine antimoniate (Glucantime^®^); ANFO: Amphotericin B. ^a^IC_50_ values to *L. (V.) braziliensisvs.* IC_50_ values to *L. (L.) infantum*, *P* > 0.05 (Unpaired t test). ^b^IC_50_ values in LIT *vs.* IC_50_ values in RPMI-1640, *P* > 0.05 (Unpaired t test).

Similar to Glucantime^®^, the CC_50_ values for compounds Pyr1 and Pyr2 were higher than the highest concentration tested (CC_50_ >400 µg/mL), suggesting low toxicity (Table 2). The analogues Pyr 4 (CC_50_ 201.7 ± 2.11 µg/mL) and Bex2 (CC_50_ 274.3 ± 1.87 µg/mL) showed both CC_50_ higher that 200 µg/mL, and the compound Bex3 has the lower cytotoxic concentration of Bexs tested, with a CC_50_ of 105.7 ± 2.26 µg/mL. All of the synthetic compounds showed lower toxicity that the drug amphotericin B, which has a CC_50_ of 89.33 ± 2.24 µg/mL (Table 2). SI values were calculated to determine the safety and efficiency of compounds. As shown in Table 2, SI values ranged from1.12 to 4.10 concerning to *L. (V.) braziliensis* and from 1.16 to 4.02 against *L. (L.) infantum.* The antileishmanial agent meglumine antimoniate (Glucantime^®^) had the highest SI value with respect to both species studied (4.10 for *L. (V.) braziliensis* and 4.02 for *L. (L.) infantum*). Thus, these results showed that the compounds more active in anti-promastigote assays has presented low toxicity against hamster spleen cells, suggesting that these analogs are relatively safe and have a wide therapeutic range.

**Table 2:** Cytotoxic concentration (CC_50_) and selective index (SI) of benzoxazinoids analogs and positive controls

**Compound**	**CC**_50_ **(µg/mL)** ^a^	**GM (µg/mL)** ^b^		**SI** ^c^	
		***L.*** ***braziliensis***	***L. infantum***	***L.*** ***braziliensis***	***L. infantum***
Pyr1(A)	>400	111.45	100.98	3.59	3.96
Pyr2 (B)	>400	102.47	114.96	3.90	3.48
Pyr4 (D)	201.7±2.11	100.47	92.98	2.01	2.17
Bex3(G)	105.7±2.26	94.47	90.98	1.12	1.16
Bex2 (H)	274.3±1.87	105.96	108.47	2.59	2.53
MEG	>400	97.5	99.47	4.10	4.02
ANFO	89.33±2.24	33.35	41.54	2.68	2.15

^a^CC_50_ is the concentration necessity for kill 50% of cells in relation to the growth control. ^b^Geometric mean (GM) of compounds IC_50_ values. ^c^Selectivity index (SI) is a ration of CC_50_ to GM of IC_50_ values. In case of CC_50_ was higher than the highest concentration tested drug, for the purpose of calculating the SI, the value used was 400. Large SI indicates a more security compound.

Jensen et al^27^ showed that Bexs obtained through the diet are widely absorbed, metabolized and excreted mainly by the urinary system. In this crossover study with 19 participants, it was shown that plasma and urinary Bex levels are proportional to the number of ingested compounds. In addition, the pharmacokinetic profile showed that they were quickly absorbed, taking approximately 3 h to reach the peak plasma levels, suggesting the plausibility Bex-derived compounds studded here being used as an oral drug.^27^ Furthermore, the secondary therapeutic effects of Bex-derived compounds may be advantageous during antileishmanial therapy, which include potential anti-inflammatory benefits. The Bexs MBOA, HMBOA and HBOA, isolated from roots of *Coixlachryma-jobi* var. *ma-yuen,* were able to reduce levels of histamine released from rat mast cells stimulated with concanavalin A and sensitized with immunoglobulin E.^28^ In addition, benzoxazinone analogues, synthesized from N-(1-(6,8-dibromo-4-oxo-4H-benzo[d][1,3]-oxazin-2-yl)-2-phenylvinyl)benzamide, showed moderate to high anti-inflammatory activity in a carrageenan-induced rat paw edema model.^29^ The anti-inflammatory effect of Bex-derived compounds is of particular relevance because some forms of leishmaniasis, such as mucocutaneous, have a crucial inflammatory component pathophysiological component.^4^ However, oral bioavailability, as well as the anti-inflammatory activity of the compounds employed in this study, have yet to be determined.

## Conclusion

Bex analogs were efficiently produced by successive nucleophilic substitution reactions followed by reductive cyclization. The parasite viability assay showed that the analogs designated Bex2, Bex3, Pyr1, Pyr2, and Pyr4 have similar levels of antileishmanial activity against the promastigotes of *L. (V.) braziliensis* and *L. (L.) infantum* as the reference drug Glucantime^®^. In addition, these compounds showed low cytotoxicity against splenic hamster cells and favorable selectivity indices. Thus, we conclude that the analogs in question are promising prototypes for the pharmaceutical industry in the development of novel, more effective and safer leishmanicidal agents.

## Ethical Issues

Not applicable

## Conflict of Interest

The authors declared no potential conflicts of interest with respect to the research, authorship, and/or publication of this article.

## Acknowledgments

W.G.L. is grateful to *Foundation for Research Support of Minas Gerais* (FAPMIG) for a graduate fellowship. J.M.S. acknowledge *National Council for Scientific and Technological Development* (CNPq) for a research grant. This work was supported by theCoordination of Improvement of Higher Level Personnel (CAPES) under Grant 2833-2011.
